# Short Sleep Duration and Insomnia Symptoms were Associated with Lower Happiness Levels in Chinese Adults in Hong Kong

**DOI:** 10.3390/ijerph16122079

**Published:** 2019-06-12

**Authors:** Sheng Zhi Zhao, Man Ping Wang, Kasisomayajula Viswanath, Agnes Lai, Daniel Yee Tak Fong, Chia-Chin Lin, Sophia Siu-Chee Chan, Tai Hing Lam

**Affiliations:** 1 School of Nursing, The University of Hong Kong, Hong Kong, SAR, China; lubabezz@hku.hk (S.Z.Z.); dytfong@hku.hk (D.Y.T.F.); cclin@hku.hk (C.-C.L.); nssophia@hku.hk (S.S.-C.C.); 2 Center for Community-Based Research, Dana-Farber Cancer Institute/Department of Social and Behavioral Sciences, Harvard University TH Chan School of Public Health, Boston, MA 02215, USA; Vish_Viswanath@dfci.harvard.edu; 3 School of Public Health, The University of Hong Kong, Hong Kong, SAR, China; agneslai@hku.hk (A.L.); hrmrlth@hku.hk (T.H.L.)

**Keywords:** sleep problems, subjective well-being, life satisfaction, Chinese, public health, population study

## Abstract

*Study objective*: To examine the association of sleep duration and insomnia symptoms with happiness. *Methods*: A random sample of 1691 Chinese adult (mean age 54 ± 20.1, male 51%) were interviewed in a population-based telephone survey. Happiness was measured by the subjective happiness scale (SHS) and the one-item global happiness index (GHI). Information on sleep included mean past seven-day sleep duration (<6 h, ≥6 to <8 h and ≥8 h) and insomnia symptoms: Difficulty in initiating sleep (DIS), difficulty in maintaining sleep (DMS), and early morning awakening (EMA). Adjusted beta-coefficient (β) of SHS and adjusted odds ratio (aOR) of GHI in relation to sleep problems were calculated. Interaction effects by age (18–65 vs. ≥65) and by sex were assessed. *Results*: Compared to ≥8 h of sleep, having <6 h of sleep had lower SHS (adjusted β −0.32, 95% CI −0.46 to −0.17) and GHI (aOR 0.54, 95% CI 0.38 to 0.78). The associations were stronger in younger adults and in women (*p* < 0.05). DIS, DMS, and EMA were associated with lower SHS (adjusted β ranged from −0.20 to −0.06) and GHI (aOR ranged from 0.57 to 0.89). Dose-response association between the number of insomnia symptoms and lower SHS was observed (*p* < 0.001). These associations were generally stronger in older adults and among women. *Conclusions*: Lower levels of happiness were observed, particularly in younger adults and females with short sleep duration and older adults and females with insomnia symptoms. Prospective studies are needed to confirm the findings and understand the mechanisms between sleep and happiness.

## 1. Introduction

Sleep is an important brain function for memory consolidation, brain reactivity and emotion regulation [1]. The prevalence for one or more insomnia symptoms is approximately 30% in adult populations in different countries [2]. Evidence suggests that insomnia may be under-diagnosed, and under-treated [3]. Sleep problems, such as short sleep duration and insomnia symptoms, were associated with overall health impairment, such as cardiovascular diseases, obesity, diabetes [4,5,6], and mental health problems, such as depression, anxiety, other psychological disorders, and suicide attempts [7]. Neurobehavioral dysregulation [3,7], emotional dysregulation [1,8,9], and well-being impairment [10] were also found to be associated with sleep problems. 

Poor well-being may be linked to sleep deprivation through neurobiological reactions, such as the amygdala hyper-limbic response, circadian rhythm disorder or hypothalamic-pituitary-adrenal axis over-activity [11,12,13]. A 60% increase in amygdala activity in response to emotional stimulus was identified under conditions of sleep deprivation [11]. In healthy adolescents, one-night sleep deprivation worsened mood states including depression, anger, confusion, anxiety, vigor, and fatigue [14]. Impaired sleep quality was found to decrease positive affect and pleasurable experience in everyday life [15]. The adverse influence on subjective life satisfaction and positive affect in people with insomnia symptoms were also reported in longitudinal studies [10]. Subjective well-being in these studies was mainly measured by relative contentment and satisfaction, which may not reflect the overall personal happiness levels [16]. The effect of sleep problems on mental health was found to be modified by age. Older subjects felt significantly worse than the young in terms of mood, tension, and physical comfort in sleep deprivation experiments [12]. Women also showed greater responses to sleep loss in terms of mood deficits [17] and wellbeing impairment [12].

Happiness, an important ingredient of well-being and health, is a state of mind or a feeling characterized by contentment, love, satisfaction, pleasure, or joy [18]. Happiness consists of personal experience [19] and is affected by various factors such as socio-economic status, dispositional factors (e.g., gene, personality), life events (e.g., marriage) and physical and mental health. We had reported that unhealthy behaviors, including smoking [20], lack of physical exercise, and alcohol drinking [21] were associated with unhappiness. Adequate sleep improves positive affect by enhanced prefrontal cortex functioning [22]. Higher sleep efficacy and fewer sleep problems were associated with happiness and positive affect in some cross-sectional studies with small sample size. [23,24]. Hong Kong is the most westernized, urbanized, and densely populated city of China. It has a high prevalence of sleep problems [25] and low happiness levels compared with other urbanized city in China (e.g., Beijing) [26]. Therefore, this study aimed to assess whether sleep duration and sleep problems would be associated with happiness levels and the moderating effects of age and gender in adult Chinese in Hong Kong.

## 2. Methods

### 2.1. Ethical Statement

Ethical approval was granted by the Institutional Review Board (IRB) of the University of Hong Kong/Hospital Authority Hong Kong West Cluster (UW 09-324). Verbal informed consents were obtained and recorded.

### 2.2. Survey Design

The Hong Kong Family and Health Information Trends Survey (FHInTS) conducted in 2016 (April to July) and 2017 (February to May) were part of the FAMILY project, which was supported by the Hong Kong Jockey Club Charities Trust. FHInTS is a regular periodic probability-based telephone survey of the general Hong Kong public, designed to assess family health, lifestyles, and physical and mental health status. Public Opinion Programme of the University of Hong Kong was commissioned to conduct the telephone interview. The target population was Hong Kong residents aged 18 or above who speak Cantonese. Details of the survey have been published elsewhere [20,27]. Briefly, using the web-based computer-assisted telephone interview system, seed numbers of residential telephone directories (cover approximately 76% of Hong Kong residents) and plus/minus one/two of the last digit of seed numbers were used to generate the sampling frame to include unlisted telephone numbers [27]. Telephone interviews were conducted by trained interviewers.

After successful contacts, eligible household residents with the birthday closest to the interview day were selected (next birthday rule) [28]. Each interview took about 25 minutes to complete. Among 11,202 people with confirmed eligibility, 8092 adults (4038 in 2016 and 4054 in 2017) were successfully interviewed yielding a response rate of 72.2%. A subset of 1691 (20.9%) respondents was randomly selected to answer questions on sleep duration, insomnia symptoms, and subjective happiness. Information about health status, health-related behaviors (diet, physical activity, alcohol, and tobacco use) and socio-demographics was also collected.

### 2.3. Measurements

Happiness was assessed by the subjective happiness scale (SHS) and global happiness item (GHI). SHS consists of four items (with the fourth item reverse-coded) with higher mean scores (1–7) represent great happiness [29]. Reliability and validity of SHS have been established in Chinese adults [30]. The global happiness item, a commonly utilized measurement of subjective happiness [31], measures subjective happiness by a single question “In general, would you say you are: Not happy at all, not very happy, happy or very happy?” GHI has been widely used in the World Wealth Survey and global happiness surveys [32].

Sleep duration was measured by self-reporting average sleeping hours in the past seven days, which was categorized into <6 h, ≥ 6 to <8 h and ≥8 h. Respondents reporting average daily sleep of less than two hours (*n* = 29) were considered outliers and excluded from the analysis [33]. Respondents were asked if they had difficulty in initiating sleep (DIS), difficulty in maintaining sleep (DMS), and early morning awakening (EMA) in the past 30 days. Insomnia symptom was defined as having any of the above three problems (yes/no). These items were frequently used to assess sleep problems in epidemiologic studies [34,35]. Our previous study has found these single-item measures of insomnia symptoms to be modestly stable over a one-month interval (Cohen’s *κ* = 0.25 to 0.48) with concurrent validity supported by significant associations with health problems, including headache, dizziness and frequent fatigue, and self-rated health [34].

Socio-demographic characteristics included age, sex, education (primary, secondary, or tertiary), household income (HK$ ≤ 20,000, > 20,000, or unstable) (HK$7.8 = US$1) and marital status (single or ever married). Health-related behaviors included smoking (ever or never), alcohol drinking (ever or never) and moderate physical activity (none, one to three days/week, or four to seven days/week), and health status such as body mass index (BMI), self-rated health, chronic disease (any or none) and mental health (any or none). Self-rated health was measured by a single question “In general, would you say your health is excellent, very good, good, fair or poor?” and was dichotomized as good or fair/poor. Respondents reported ever diagnosed with any chronic diseases such as high cholesterol, hypertension, heart disease, or coronary heart disease, stroke, diabetes, cancer, hepatitis, arthritis, bronchitis, asthma or allergic rhinitis. Mental health was assessed by patient health questionaire-4 (PHQ-4) scale, a four-item valid measure of anxiety and depression symptom [36]. The scale is reliable (Cronbach’s α = 0.85), and the internal consistency was also satisfactory in our sample (α = 0.82).

### 2.4. Analysis

The data were analyzed using STATA 13.1. To reduce the bias of our estimators and ensure the representativeness of the findings, all data were weighted by the gender-age distribution of the Hong Kong population in 2015 year-end and the educational attainment (highest level attended) distribution collected in the 2011 Census. Adjusted beta-coefficient (β) of SHS by linear regression was used to examine its association with sleep duration and insomnia symptoms (DIS, DMS, and EMA). Sex, age, education, household income, marital status, smoking, alcohol drinking, physical activity, self-report health, and chronic disease were adjusted in the regression model 1. Model 2 additionally adjusted for mental health status (depression and anxiety symptoms). Generalized ordered logistic regression was used to calculate the adjusted odds ratio (aOR) of GHI [37]. *p* < 0.05 was considered as statistical significance. Effect modifications by age (18–65/65+ years old) and sex on the associations between sleep problems and happiness (SHS) were analyzed using the interaction terms.

## 3. Results

Table 1 shows that of the 1691 respondents, after weighting, 51% were male, 85.3% were younger adults (aged 18 to below 65 years), and 14.7% were older adults (aged 65 years or above) with the mean age of sample 54 ± 20.1. Additionally, 29.8% were single. Most respondents (74.8%) had secondary or higher education attainment, and 58.9% had a monthly household income of HK $20,000 or more (the median monthly household income in Hong Kong was $25,000 in 2016 [38]). Around a quarter (24.8%) of the respondents were ever-smokers and half (51.5%) were ever-drinkers. More than half (63.8%) were physically inactive, and 42.9% were overweight or obese (BMI ≥ 23 in Asians). Half (51.8%) reported poor or fair self-rated health, and around one-third (31.1%) had a history of diagnosed chronic disease. Using the PHQ-4, 14.1% of the respondents were rated as having poor mental health (with anxiety or depression symptoms). The mean scores were 5.0 (± 1.0) for SHS and 3.0 (± 0.6) for GHI. The mean sleep duration was 6.7 (± 1.4) hours, and 17.2% slept for less than six hours. Over half (61.2%) had a least one insomnia symptom: 33.5% had DIS, 47.1% had DMS, and 30.1% had EMA. 

Compared with ≥8-h sleep, ≥6 to <8 h and <6-h sleep was associated with adjusted β (95% CI) of −0.15 (−0.26 to −0.04) and −0.38 (−0.53 to −0.23) for SHS, respectively. The corresponding aORs (95%CI) for GHI were 0.76 (0.58 to 0.99) and 0.49 (0.34 to 0.70) (*p* < 0.001) (Table 2). Per hour sleep reduction was associated with lower SHS (adjusted β 0.09, 95% CI −0.12 to −0.05) and GHI (aOR 0.76, 95% CI 0.66 to 0.87). DIS, DMS and EMA were associated with lower SHS (adjusted β ranged from −0.26 to −0.08) and GHI (aOR ranged from 0.52 to 0.86), all *p* < 0.001. Any insomnia symptom was associated with both lower SHS (adjusted β −0.25, 95% CI −0.35 to −0.15) and GHI (aOR 0.59, 95% CI 0.46 to 0.75) with dose-response relationship for the number of symptoms (*p* < 0.001). Per insomnia symptom increase was associated with lower SHS (adjusted β 0.14, 95% CI −0.18 to −0.10) and GHI (aOR 0.74, 95% CI 0.66 to 0.82). The results remained significant in models additionally adjusted for mental health status (model 2). 

The association of shorter sleep duration and more insomnia symptoms with lower SHS was modified by age and sex (Table 3). Shorter sleep duration was associated with lower SHS in younger adults (adjusted β −0.38, 95% CI −0.39 to −0.11) but not older adults (adjusted β −0.16, 95% CI −0.26 to 0.14) (*p* < 0.01). In contrast, the association of DMS and the number of insomnia symptoms with SHS was stronger in older than younger adults (*p* < 0.05). Similar associations were observed in both groups for the associations of EMA and any insomnia symptoms with SHS (*p* > 0.11). DIS was significantly associated with lower SHS in young (adjusted β −0.23, 95% CI −0.35 to −0.11) but not old (adjusted β −0.15, 95% CI −0.31 to 0.01) adults. Women had stronger associations of shorter sleep duration (adjusted β ranged from −0.19 to −0.39), number of insomnia symptoms (adjusted β ranged from −0.15 to −0.45), DMS (β −0.00 vs. −0.13) and EMA (β 0.01 vs. −0.09) with SHS (all *p* < 0.05).

## 4. Discussion

We provide arguably the first evidence on the associations of shorter sleep duration and insomnia symptoms with lower happiness using a population representative sample. The results were robust using two different measures of happiness and after adjusting for various confounders. Our findings were in line with other psychological studies showing a negative association of sleep problems with mental health and well-being [3,10]. Daily cognitive impairment disrupted circadian rhythm, emotional fluctuation, and lack of positive emotion experienced due to sleep problems might lower happiness levels [1,39]. The robust associations between sleep problems and happiness independent of mental health in our study suggested other potential pathways, such as tiredness, fatigue, and physical discomfort due to the poor sleep. The associations can also be explained by late night sleep or insufficient sleep related to other unhealthy lifestyles, such as internet addiction or problematic smartphone use, which was associated with reduced daytime vitality and lowered happiness levels [40,41].

The dose-response associations of sleep duration and the number of insomnia symptoms with lower happiness levels in our study were in concordance with experimental studies on the effects of sleep deprivation on neurobehavioral function impairment and accumulative cognitive deficits [42]. A meta-analysis of cross-sectional, case-control, and cohort study also reported that the risk of suicide attempts decreased in a dose-response manner by 11% for the hourly increase in sleep duration [43]. Our findings raised the alarm of sleep deprivation which is prevalent in many metropolitan cities, including Hong Kong, where many people (17.2%) sleep for less than six hours [25], which is lower than the suggested duration of sleep (seven to nine hours for younger adults and seven to hours for older adults) [44]. Nearly 40% of Hong Kong adults (39.4%) [25] had insomnia symptoms, which was also greater than those in the west (30%). [2] Raising public awareness of sleep problems on subjective happiness may help promote the need for better sleep hygiene [45].

The happiness levels in older adults appeared less vulnerable to short sleep duration. This may be explained by the reduced biological need for sleep, as they reported less subjective sleepiness and a smaller increase in lapses of attention following sleep restrictions [46,47]. Older adults are mostly retired and could afford more naps than younger adults to compensate for the negative effect of short sleep duration [44]. The dose-response associations of insomnia symptoms with reduced happiness were stronger in older adults, because they may be more easily awoken by external sensory stimuli (i.e., greater sleep fragility) [44,46], and may have greater mood disturbance after sleep loss [12]. Evidence suggests that increased DMS was associated with increased age and once awakened during sleep, older adults took longer to return to sleep [48], which in turn led to a more severe sleep loss in the older adults leading to lower happiness. Nevertheless, the deterioration in overall health status in some individuals could also result in the significantly stronger effect on lower happiness particularly when having more insomnia symptoms (e.g., having three symptoms).

The stronger association between sleep problems and lowered happiness in females were in line with sleep deprivation experiments in which sleep problems predicted mood deficit, emotional dysregulation, well-being impairment, and mental disease more significantly in women than men [12,17,49]. The sex-specific association of sleep problems on lowered happiness levels may be explained by the changes in the endocrine control of the reproductive system occurring during the menstrual cycle, pregnancy, after parturition, and during the menopause in females [49,50]. An increased risk of mood disturbances related to poor sleep during the periods and a heightened sensitivity to hormone changes on the propensity for mood dysregulation could be part of the mechanism [17,49]. Studies also suggested an inherited gender-specific vulnerability explained by the higher depression incidence in women [49,50]. Women also face a greater burden of discrimination, social inequality, conflict between work and family roles and fertility responsibilities that might have contributed to the emotional fluctuation in response to sleep loss and lead to lowered happiness levels. 

Our findings, if confirmed by prospective studies, can provide important clinical and public health implications to increase awareness of the benefit of good sleep hygiene and improve subjective happiness in the general population. Integrated with extensive research on sleep and quality of life, this study emphasizes the importance of regular evaluation of sleep complaints by health professionals with a referral for behavioral or pharmacological interventions. Interventions to increase the level of happiness, such as daily gratitude exercises [51] or behavior interventions (smoking cessation, a decrease in alcohol consumption, and an increase in physical activity) [20,21] were important in reducing the negative effects of poor sleep experience.

### Limitations

This study had several limitations. First, varying criteria and definitions of insomnia were used in previous sleep studies. We have used simple measurements of sleep duration and sleep problems which have been widely used in population-based studies [34,35]. Objective measures, such as actigraphy and polysomnography (with an electroencephalogram (EEG)) would offer more valid results, but the implementation is not feasible in a sample of this size. Second, bidirectional association is possible in which unhappiness (for example, due to psychological stress) may predict poor sleep. Although reverse causality cannot be excluded from cross-sectional studies, our results may be explained by underlying neurobiological mechanisms, such as abnormalities in circadian rhythms and overactivity of the hypothalamic-pituitary-adrenal axis caused by sleep problems which are closely linked to emotion dysregulation, poor well-being, and unhappiness [8,12,13]. Third, we had adjusted for many potential confounders, but residual confounding effects (e.g., personality trait) cannot be ruled out.

## 5. Conclusions

We found robust associations of short sleep duration and insomnia symptoms with lower happiness levels in a representative sample of Hong Kong Chinese adults. Lower levels of happiness were observed, particularly in younger adults and females with short sleep duration and older adults and females with insomnia symptoms. Prospective studies are needed to confirm the findings and understand the mechanisms between sleep and happiness. 

## Figures and Tables

**Table 1:** Sociodemographic characteristics, behaviors, mental health and sleep problems.

		*n* (%)	Weighted *n* (%)
Male		661 (39.0)	862 (51.0)
Age, years	18–24	210 (12.4)	143 (8.5)
	25–44	280 (16.6)	690 (40.5)
	45–64	601 (35.5)	610 (36.3)
	65+	600 (35.5)	249 (14.7)
Education	Primary or below	365 (21.6)	436 (25.8)
	Secondary	739 (43.7)	844 (49.9)
	Tertiary or above	587 (34.7)	421 (24.9)
Marital status (single)		398 (23.5)	504 (29.8)
Household income (HK$) *	≤20,000	694 (41.0)	626 (37.1)
	>20,000	934 (55.2)	996 (58.9)
	Unstable	63 (3.7)	68 (4.0)
Ever smoking		303 (17.9)	419 (24.8)
Ever alcohol use		845 (49.9)	871 (51.5)
Moderate physical activity	None	1054 (62.4)	1079 (63.8)
	1–3 days/week	308 (18.2)	331 (19.6)
	4–7 days/week	327 (19.4)	281 (16.6)
Body mass index (BMI)	<18.5	237 (14.5)	224 (13.3)
	18.5–22.9	711 (43.5)	742 (43.9)
	23–24.9	312 (19.1)	294 (17.4)
	≥25	374 (22.9)	431 (25.5)
Poor or fair self-rated health		864 (51.1)	876 (51.8)
Any chronic disease		675 (39.9)	526 (31.1)
Poor mental health		211 (12.5)	238 (14.1)
Sleep duration	≥8 h	409 (24.6)	386 (22.8)
	≥6 to <8 h	954 (57.6)	994 (60.0)
	<6 h	294 (17.7)	285 (17.2)
Mean sleep duration (SD), hours	6.65 (1.35)		
Insomnia	DIS	633 (37.4)	566 (33.5)
	DMS	829 (49.0)	796 (47.1)
	EMA	532 (31.5)	509 (30.1)
	DIS, DMS or EMA	1083 (64.0)	1034 (61.2)

DIS: Difficulty in initiating sleep, DMS: Difficulty in maintaining sleep, EMA: Early morning awakening, * US$1.00 = HK$7.80.

**Table 2:** Associations of sleep problems with happiness.

	SHS Happiness (Mean 5.03, SD 1.04)		GHI Happiness (Mean 3.00, SD 0.55)	
	Model 1 ^a^ β (95% CI)	Model 2 ^b^ β (95% CI)	Model 1 ^a^ OR (95% CI)	Model 2 ^b^ OR (95% CI)
**Sleep duration**				
≥8 h	0	0	1	1
≥6 to <8 h	−0.15 (−0.26 to −0.04) **	−0.14 (−0.25 to −0.03) *	0.76 (0.58 to 0.99) *	0.78 (0.59 to 1.02)
<6 h	−0.38 (−0.53 to −0.23) ***	−0.32 (−0.46 to −0.17) ***	0.49 (0.34 to 0.70) ***	0.54 (0.38 to 0.78) ***
*p* for trend	<0.001	<0.001	<0.001	<0.001
Per hour decrease	−0.09 (−0.12 to −0.05) ***	−0.07 (−0.10 to −0.03) ***	0.76 (0.66 to 0.87) ***	0.79 (0.67 to 0.91) ***
**Insomnia symptoms**				
DIS	−0.26 (−0.36 to −0.16) ***	−0.20 (−0.29 to −0.10) ***	0.52 (0.41 to 0.66) ***	0.57 (0.4675 to 0.74) ***
DMS	−0.10 (−0.15 to −0.05) ***	−0.08 (−0.13 to −0.04) ***	0.82 (0.73 to 0.92) ***	0.84 (0.75 to 0.95) **
EMA	−0.08 (−0.11 to −0.04) ***	−0.06 (−0.10 to −0.03) ***	0.86 (0.80 to 0.94) ***	0.89 (0.82 to 0.97) **
Any of these symptoms	−0.25 (−0.35 to −0.15) ***	−0.18 (−0.28 to −0.09) ***	0.59 (0.46 to 0.75) ***	0.66 (0.51 to 0.85) ***
Number of insomnia symptoms				
0	0	0	1	1
1	−0.15 (−0.26 to −0.03) *	−0.10 (−0.21 to 0.02)	0.77 (0.58 to 1.03)	0.85 (0.64 to 1.14)
2	−0.24 (−0.37 to −0.11) ***	−0.18 (−0.31 to −0.05) **	0.54 (0.39 to 0.74) ***	0.59 (0.43 to 0.82) **
3	−0.44 (−0.59 to −0.30) ***	−0.35 (−0.49 to −0.21) ***	0.41 (0.29 to 0.58) ***	0.50 (0.35 to 0.71) ***
Per symptoms increase	−0.14 (−0.18 to −0.10) ***	−0.11 (−0.15 to −0.07) ***	0.74 (0.66 to 0.82) ***	0.78 (0.69 to 0.87) ***

SHS: Subjective happiness scale, GHI: Global happiness index, DIS: Difficulty in initiating sleep, DMS: Difficulty in maintaining sleep, EMA: Early morning awakening, * *p* < 0.05. ** *p* < 0.01. *** *p* < 0.001. ^a^ Model 1: Adjusted for sex, age, education, smoking, alcohol, moderate physical activity, body mass index (BMI), household income, marital status, self-rated health, and chronic disease. ^b^ Model 2: Adjusted for model 1 variables and mental health symptoms.

**Table 3:** Moderating effects of age and sex on the associations between sleep problems and happiness (SHS).

	Age		Interaction Term ^a^	Sex		Interaction Term ^a^
	Adjusted β ^b^		*p*-Value	Adjusted β ^c^		*p*-Value
	18–65 Years Old (*n* = 1091)	65+ Years Old (*n* = 600)		Male (*n* = 660)	Female (*n* = 1031)	
**Sleep duration**			0.003			<0.001
≥8 h	0	0		0	0	
≥6 to <8 h	−0.20 (−0.33 to −0.07) **	0.03 (−0.17 to 0.23)		−0.05 (−0.23 to 0.13)	−0.19 (−0.33 to −0.05) **	
<6 h	−0.38 (−0.57 to −0.20) ***	−0.16 (−0.40 to 0.07)		−0.19 (−0.43 to 0.05)	−0.39 (−0.57 to −0.20) ***	
*p* for trend	<0.001	0.18		0.13	<0.001	
Per hour decrease	−0.08 (−0.13 to −0.04) ***	−0.04 (−0.10 to 0.01)		−0.04 (−0.10 to 0.02)	−0.08 (−0.13 to −0.04) ***	
**Insomnia symptoms**						
DIS	−0.23 (−0.35 to −0.11) ***	−0.15 (−0.31 to 0.01)	0.97	−0.20 (−0.36 to −0.04) *	−0.19 (−0.31 to −0.07) **	0.94
DMS	−0.05 (−0.11 to 0.01)	−0.14 (−0.22 to −0.06) ***	0.01	−0.00 (−0.08 to 0.07)	−0.13 (−0.19 to −0.08) ***	0.007
EMA	−0.04 (−0.08 to −0.00) *	−0.08 (−0.14 to −0.03) **	0.11	0.01 (−0.07 to 0.04)	−0.09 (−0.13 to −0.05) ***	0.02
Any of these symptoms	−0.17 (−0.28 to −0.05) **	−0.22 (−0.39 to −0.05) *	0.18	−0.09 (−0.24 to 0.07)	−0.26 (−0.38 to −0.13) ***	0.08
Number of insomnia symptoms			<0.001			<0.001
0	0	0		0	0	
1	−0.09 (−0.23 to 0.04)	−0.11 (−0.32 to 0.09)		−0.05 (−0.22 to 0.13)	−0.15 (−0.30 to 0.00)	
2	−0.22 (−0.38 to −0.06) **	−0.11 (−0.32 to 0.11)		−0.12 (−0.33 to 0.08)	−0.23 (−0.39 to −0.06) **	
3	−0.25 (−0.43 to −0.08) **	−0.48 (−0.71 to −0.26) ***		−0.13 (−0.38 to 0.11)	−0.45 (−0.62 to −0.28) ***	
Per symptoms increase	−0.09 (−0.15 to −0.04) ***	−0.14 (−0.21 to −0.07) ***		−0.05 (−0.12 to 0.02)	−0.14 (−0.19 to −0.09) ***	

^a^ Model 2: Adjusted for socio-demographic characteristics, health-related behaviors, physical health, and mental health symptoms. ^b^ Adjusted for sex, education, household income, marital status, health-related behaviors, physical and mental health symptoms. ^c^ Adjusted for age, education, household income, marital status, health-related behaviors, physical, and mental health symptoms. DIS: Difficulty in initiating sleep, DMS: Difficulty in maintaining sleep, EMA: Early morning awakening. * *p* < 0.05, ** *p* < 0.01, *** *p* < 0.001.
